# *KRAS* mutation: The booster of pancreatic ductal adenocarcinoma transformation and progression

**DOI:** 10.3389/fcell.2023.1147676

**Published:** 2023-04-20

**Authors:** Zining Zhang, Heng Zhang, Xiang Liao, Hsiang-i Tsai

**Affiliations:** ^1^ Institute of Medical Imaging and Artificial Intelligence, Jiangsu University, Zhenjiang, China; ^2^ Department of Medical Imaging, The Affiliated Hospital of Jiangsu University, Zhenjiang, China

**Keywords:** PDAC, KRAS mutation, phosphokinase, metabolic reprogramming, therapy resistance, poorer prognosis

## Abstract

Pancreatic ductal adenocarcinoma (PDAC) is the most common type of pancreatic cancer. It has a poor response to conventional therapy and has an extremely poor 5-year survival rate. PDAC is driven by multiple oncogene mutations, with the highest mutation frequency being observed in *KRAS*. The KRAS protein, which binds to GTP, has phosphokinase activity, which further activates downstream effectors. *KRAS* mutation contributes to cancer cell proliferation, metabolic reprogramming, immune escape, and therapy resistance in PDAC, acting as a critical driver of the disease. Thus, *KRAS* mutation is positively associated with poorer prognosis in pancreatic cancer patients. This review focus on the *KRAS* mutation patterns in PDAC, and further emphases its role in signal transduction, metabolic reprogramming, therapy resistance and prognosis, hoping to provide *KRAS* target therapy strategies for PDAC*.*

## 1 Introduction

The incidence of pancreatic cancer is increasing at a rate of 0.5%–1.0% per year, and pancreatic cancer is expected to be the second leading cause of cancer death by 2030. Pancreatic cancer includes pancreatic ductal adenocarcinoma (PDAC), which accounts for 90%, follicular carcinoma, pancreatic blastoma, and neuroendocrine tumors. The malignancy of pancreatic cancer is high and the 5-year survival rate of PDAC is as low as 10% (Park et al., 2021). Most PDAC patients fail to be cured by radical surgery due to diagnosis at late or advanced disease stages. Current chemotherapy avenues only prolong the survival of PDAC patients by a few months. Although advanced research tools, such as pancreatic duct-like organs (PDLOs) and pancreatic cancer patient-derived organoids (PDOs), provide unique advantages for developing precise medicines and novel drugs, targeted therapies, including immune therapies, for PDAC are still limited (Melzer et al., 2022). Thus, it is important to develop effective therapies for PDAC (Bear et al., 2020).

PDAC is characterized by a high (up to 95%) mutation rate in the Kirsten rat sarcoma virus oncogene homolog (*KRAS*) gene. *KRAS* signaling is essential for PDAC progression, as it regulates multiple downstream effectors. Moreover, *KRAS* signaling can remodel the PDAC tumor microenvironment (Eser et al., 2014; Hou et al., 2020; Ohara et al., 2022).Previously, *KRAS* signaling leading to PDAC was considered to be “non-druggable” (Ferguson et al., 2022). Strikingly, there have been several recent advances in the direct targeting of *KRAS*, particularly with *KRAS* ^*G12C*^ inhibitors. Most existing *KRAS* inhibitors, such as AMG510 (sotorasib) and MRTX849 (adagrasib), have shown effect in non-small cell lung cancer treatment (Tang et al., 2021), with limited effect on PDAC. Although there are many therapeutic approaches targeting *KRAS* mutations, few are truly effective in suppressing the effects of *KRAS* mutations and related pathways in PDAC (Choi et al., 2019). Moreover, translation of the few promising results obtained through research to clinical application has been very disappointing.

## 2 *KRAS* mutation in PDAC

PDAC is the most common pancreatic cancer. It involves the progressive differentiation of normal pancreatic ductal epithelium to precancerous lesions, such as pancreatic intraepithelial neoplasia (PanIN). The latest clinical statistics from Chinese PDAC patients revealed recurrent somatic mutations, including *KRAS* (83.2%), *TP53* (70.6%), *CDKN2A* (28.8%), *SMAD4* (23.0%), *ARID1A* (12.8%), and *CDKN2B* (8.9%) mutations (Zhang et al., 2022), with the highest percentage of mutations being observed in *KRAS* (Deramaudt and Rustgi, 2005). KRAS includes 150 structural domains, of which 144 can be resolved using *X*-ray crystallography and the remaining using NMR data-driven models. Among all known KRAS structures, eight are hypervariable region (HVR) peptides (flexible C-terminal structural elements), and the remaining 142 structures form a *G* domain (containing six *β* chains and five *α* helices) (Pantsar, 2020).

**FIGURE 1:**
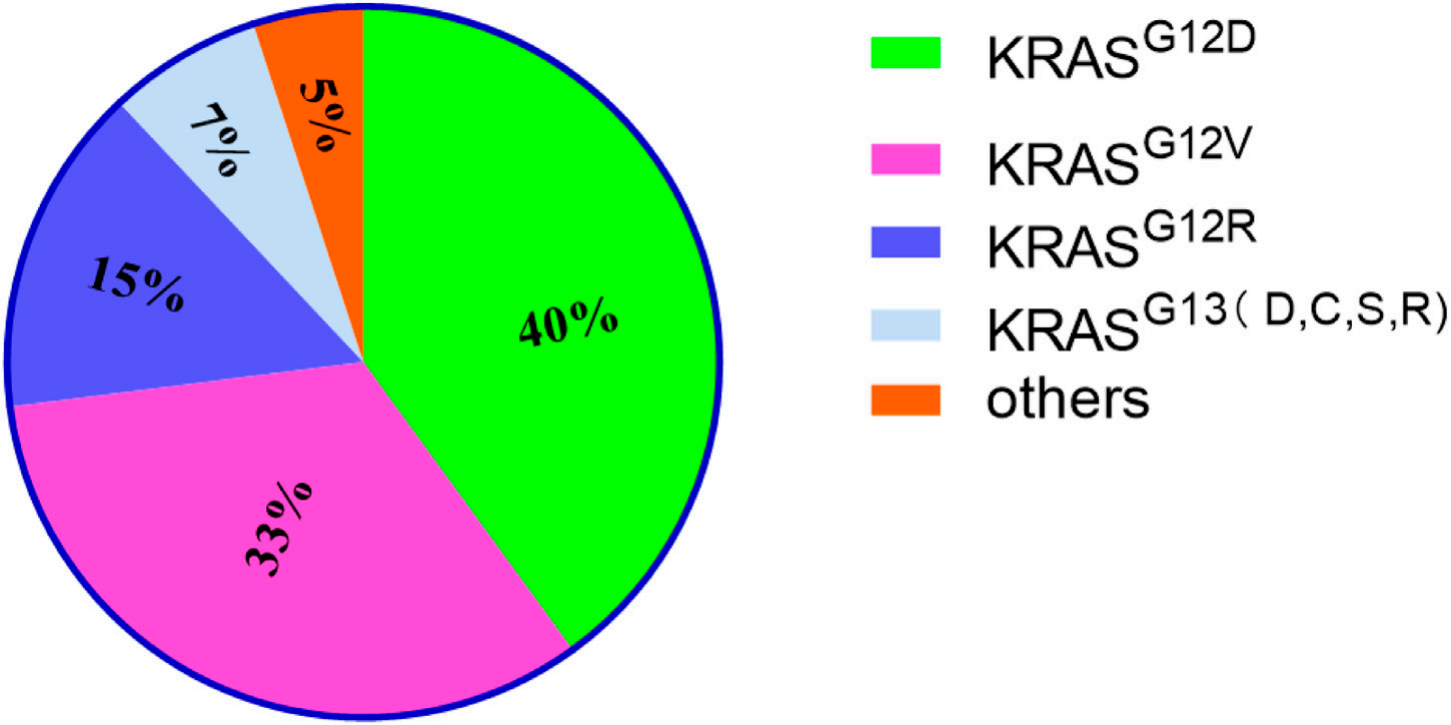
The proportion of KRAS mutations of different types. It mainly lists 4 types and others. Among them, KRAS^G12D^ accounts for the highest proportion.

The RAS family of proteins, encoded by the highly homologous genes *HRAS*, *NRAS*, and *KRAS*, is the most common protein family, the genes of which (most importantly *KRAS*) are mutated in human cancer (Hamarsheh et al., 2020). Moreover, the *KRAS* mutation-activated signaling pathway could stimulate the expression of HRAS and NRAS proteins via the induction of eNOS and C118 expression to promote tumor growth (Counter, 2015). The most common mutation in *KRAS* occurs at codon 12 of the second exon, on the first or second nucleotide, resulting in a conformational change of the GTP-binding site and, thereby, reducing the intrinsic rate of GTP hydrolysis (Neuzillet et al., 2014). *KRAS* mutations mainly occur in GGT sequences, namely GAT (G12D, 40%), GTT (G12V, 33%), CGT (G12R, 15%), TGT (G12C), GCT (G12A) and AGT (G12S) (Zhang et al., 2022). Other *KRAS* mutations occur at codon 13 of the second exon (G13D, G13C, G13S and G13R) (7%), codon 61 of the third exon (Q61H, Q61R, Q61K and Q61L), codon 117 and 146 of the fourth exon (K117 and A146) (Buscail et al., 2020) (Figure 1). Additionally, point mutations in codons 12 and 13 of the second exon of *KRAS* have been identified in the sera of some pancreatic cancer patients (Nakano et al., 2018). Patients carrying KRASG12D have a worse prognosis (Valsangkar et al., 2015).

## 3 Regulation of signaling pathways in PDAC by *KRAS* mutation

**FIGURE 2:**
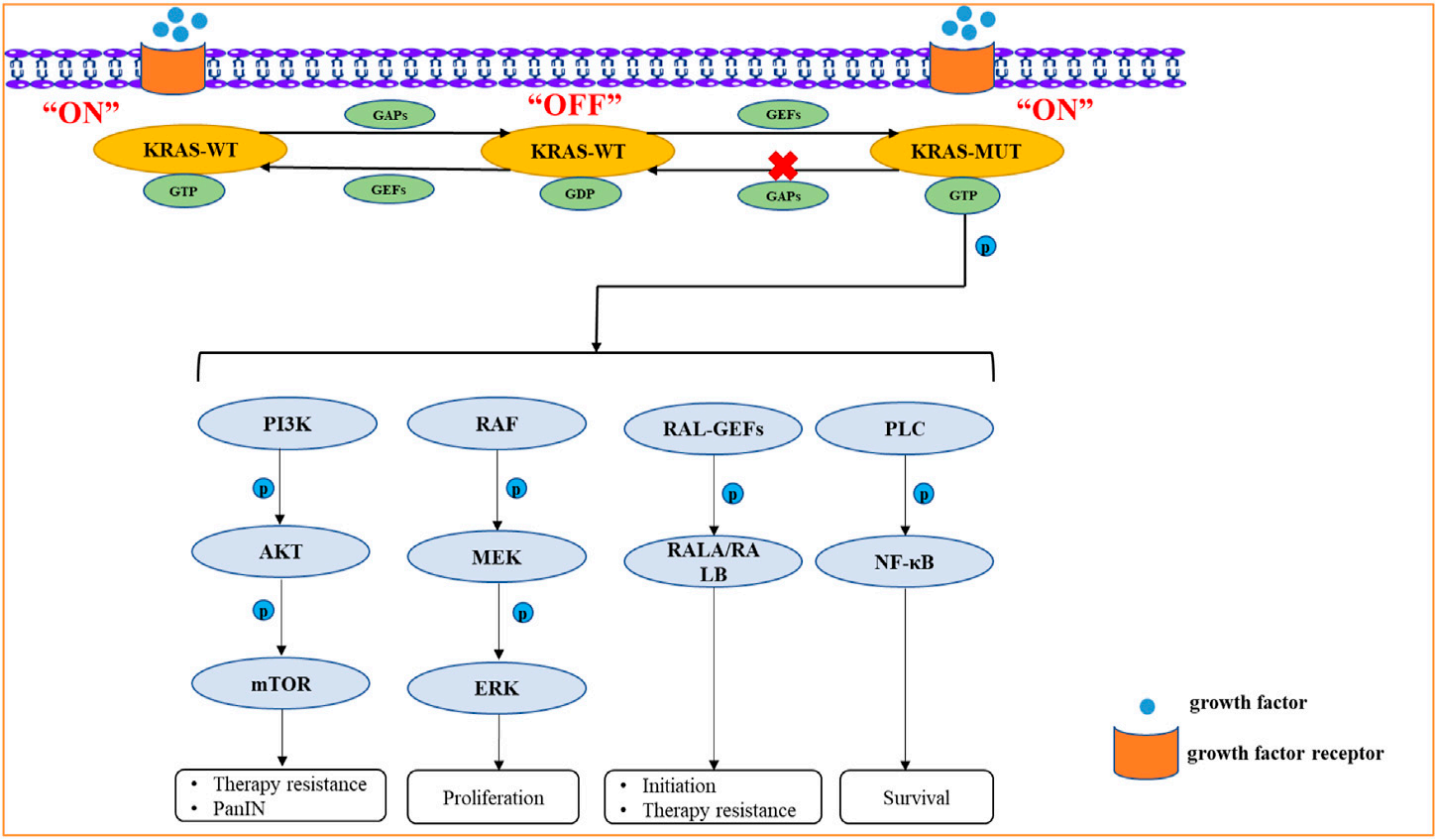
The signaling pathways activated by *KRAS* mutation include the RAF/MEK/ERK, PI3K/AKT/mTOR, RAL-GEFs, PLC signaling pathways. *KRAS* mutation can activate these signaling pathways by Phosphorylation. Due to the greatly reduced hydrolysis rate of GTP, it retains its active GTP binding state for an unusually long time. The KRAS protein, which binds to GTP, has phosphokinase activity and represents the “ON” state, which further activates downstream proteins.

*KRAS* mutation plays an important role in the initiation, development, proliferation, metastasis, metabolic reprogramming, and therapy resistance of PDAC. *KRAS* protein, which binds to GTP, having phosphokinase activity can activate various signaling pathways, such as the mitogen-activated protein kinase (MAPK), phosphatidylinositol 3-kinase (PI3K), RAL guanine nucleotide exchange factors (RAL-GEFs) and PLC pathways (Martins et al., 2014; Hamarsheh et al., 2020). The more classical signaling pathways activated by *KRAS* mutation include the RAF/MEK/ERK and PI3K/AKT/mTOR pathways (Mann et al., 2016; Ponz-Sarvise et al., 2019). KRAS/ERK signaling pathway mainly leads to the therapy resistance of PDAC, such as gemcitabine (Principe et al., 2022).In addition, the activation of ERK causes PanIN (Neuhöfer et al., 2021). KRAS/AKT signaling pathway can stimulate cellular nutrient uptake, energy production, regulation of cell cycle and apoptosis and synthesis of critical molecules (Ebrahimi et al., 2017; Dibble et al., 2022). KRAS/RAL-GEFs signaling pathway is crucial for the initiation and therapy resistance of PDAC (Seguin et al., 2014). KRAS/PLC signaling pathway can govern the survival of tumor cells (Buscail et al., 2020) (Figure 2).

## 4 *KRAS* mutation in PDAC development and progression

### 4.1 Involvement of *KRAS* mutation in the development of PDAC

**FIGURE 3:**
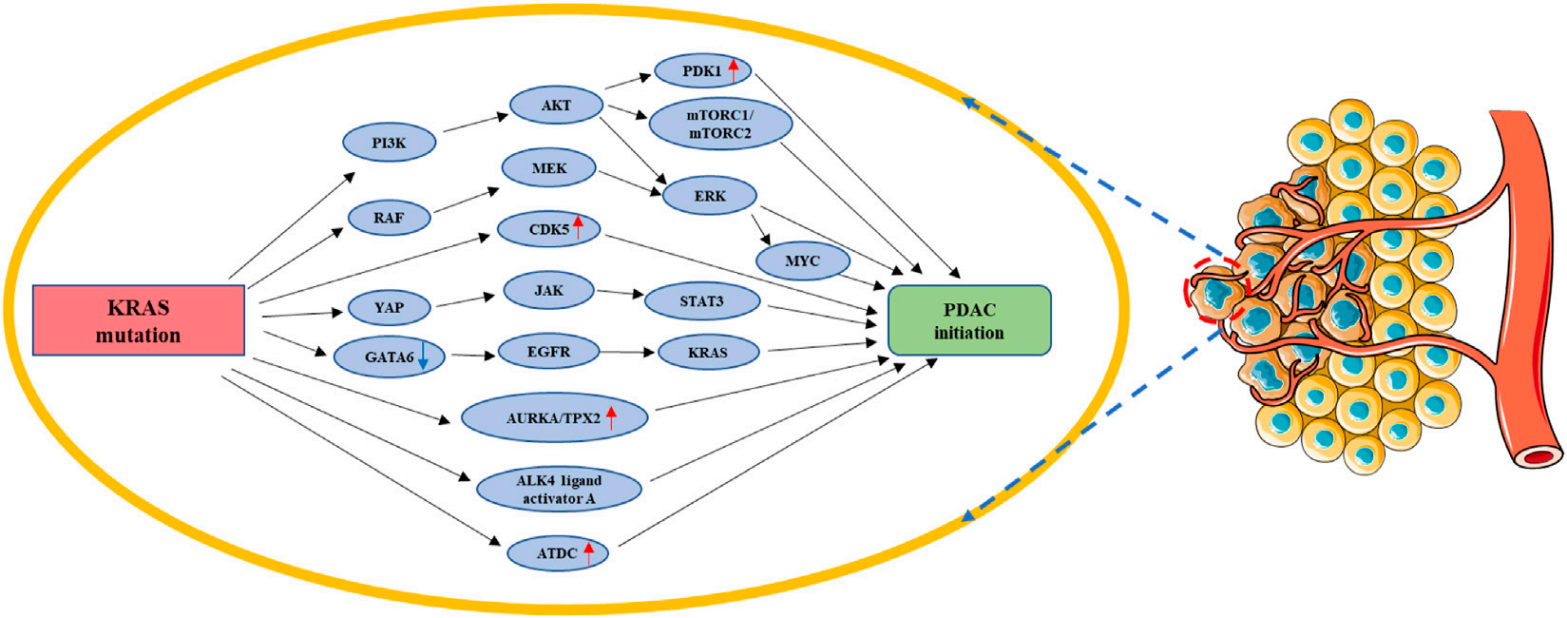
*KRAS* mutation in PDAC initiation. *KRAS* mutation could facilitate the transformation of acinar cells or ductal cells into pancreatic cancer cells by regulating PI3K/AKT/(PDK1&mTORC1/2&ERK-MYC), RAF/MEK/ERK, CDK5, YAP/JAK/STAT3, AURKA/TPX2, ALK4 ligand activator A, ATDC, and GA-TA6/EGFR/*KRAS*. Red arrow: The expression of related gene is upregulated. Blue arrow: The expression of related gene is downregulated.

*KRAS* mutation facilitates the transformation of acinar cells or ductal cells into pancreatic cancer cells (Flowers et al., 2021). Using a 3D cell culture system, the activation of RAF/MEK/ERK signaling, which is critical for acinus to duct metaplasia (ADM) progression, was studied in pancreatic acinar cells with *KRAS* ^*G12D*^ mutation (Shi et al., 2013). In the *SST*22+/- pancreatic cancer mouse model, sustained PI3K-AKT signaling activation could cross with *KRAS* ^*G12D*^ mice and further accelerate PDAC progression (Chalabi-Dchar et al., 2015). Using genetic and pharmacological methods, Ferro, R. & Falasca, M identified the *KRAS* ^*G12D*^/*PI3K*/*PDK1* signaling pathway as a critical driver of ductal chemotaxis, PanIN transformation, and PDAC maintenance (Ferro and Falasca, 2014). Amplification of the *CDK5* (cell cycle protein 5) gene or its major activators, *p35* and *p39*, was observed in 67% of PDAC cases. Compared to the wild-type *KRAS*-mutation containing human pancreatic cancer cells, human pancreatic cancer cells containing the *KRAS* ^*G12D*^ mutation displayed an increased CDK5 kinase activity and enhanced *p35* cleavage, which contribute to early stage PDAC progression (Eggers et al., 2011). Both mTORC1 and mTORC2 were significantly activated in human and mouse ADM lesions and synergistically contributed to *KRAS* ^*G12D*^-driven ADM *in vitro* and in a pancreatic carcinoma mouse model (Zhao et al., 2021). The *KRAS* ^*G12D*^-activated RAF/MEK/ERK signaling pathway often acts synergistically with AKT, which is essential for PDAC initiation and progression (Collisson et al., 2012). In an observation study of pancreatic cancer initiation and progression among smokers, it was found that nicotine promoted the development of KRAS mutation-induced pancreatic cancer, which reduced the expression of GATA6 by activating the AKT-ERK-MYC signaling pathway, which is essential for accelerating PDAC development (Hermann et al., 2014). While assessing the relationship between *KRAS* mutation and inflammation, Zhang, W. et al. found that environmental factors can enhance *KRAS* mutation and, further, activate YAP-TAZ-JAK-STAT3 signaling, which is required for the development of ADM (Gruber et al., 2016). Pancreatitis could induce *KRAS* mutation synergistically with NOTCH signaling, and these mutations could further promote the conversion of PanIN into PDAC (De La and Murtaugh, 2009). While exploring potential therapeutic targets for PDAC, *Gomes-Filho, S. M*. *et al.* found that *KRAS* mutation could upregulate *AURKA* and *TPX2* in PDAC cells to promote rapid PDAC cell proliferation (Gomes-Filho et al., 2020). *KRAS* mutation can lead to the spontaneous loss of *GATA6*, which may result in pancreatic acinar differentiation program *GATA6* dysregulation and also upregulate epidermal growth factor (EGFR), an upstream activator of *KRAS*; this *KRAS*-*GATA6*-*EGFR*-*KRAS* positive loop could significantly boost PDAC initiation and progression (Martinelli et al., 2016). It has also been reported that *KRAS* ^*G12D*^-activated *EGFR* signaling enhanced *Sox9* transcription and further promoted follicular-to-ductal transformation in mouse pancreatic tissue (Chen et al., 2015). *KRAS* mutation can inhibit the senescence of primary pancreatic ductal cells and maintain these cells in a permanent growth arrest state, which could lead to tumorigenesis under certain conditions (Lee and Bar-Sagi, 2010). By integrating genomics, single-cell chromatin analysis, and spatiotemporally controlled functional perturbations in an *in situ* mouse model, one study revealed that *KRAS* mutation may accelerate the malignant transformation of damaged pancreatic tissue (Alonso-Curbelo et al., 2021). Moreover, a study indicated that the Sox9-regulated formation of precancerous lesions and their development into tumors are related to *KRAS* mutation (Kopp et al., 2012). *KRAS* ^*G12D*^ could also interact with GNASR201C, a key pancreatic cancer maintenance factor, to promote the initiation of intraductal papillary mucinous neoplasia (IPMN) and, thereby, IPMN-induced PDAC (Patra et al., 2018). SMAD4, a central mediator of TGF-β signaling, is specifically inactivated in more than half of PDAC cases, but the loss of SMAD4 expression in itself does not promote PDAC development and needs the presence of *KRAS* mutation (Zhao et al., 2018). Transcription activator factor 3 (ATF3) could activate the unfolded protein response (UPR) and promote the formation of ADM in pancreatitis in the presence of *KRAS* ^*G12D*^ (Azizi et al., 2021). In addition, *KRAS* ^*G12D*^ could activate enhancer networks in progenitor cells associated with pancreatitis to drive PDAC development (Li et al., 2021a). Histological analysis of human and mouse tumor tissues revealed that ALK4 drives PDAC formation through *KRAS* ^*G12D*^-induced ALK4 ligand activator A upregulation in transformed glandular follicle cells (Zhao et al., 2020). *KRAS* ^*G12D*^ was also found to upregulate the ataxia capillaris group *D* complementary gene (ATDC) in PanIN subpopulations and accelerate PanIN progression in a mouse model (Wang et al., 2015). To determine whether the viral oncoprotein *SV40* large T antigen (TAg) can promote PDAC in association with *KRAS* mutations, investigators used *KRAS* ^*G12D*^ wide type and knockdown mouse models; they found that Tag could cause the development of highly invasive adenocarcinoma in the short term only in the presence of *KRAS* ^*G12D*^ (Yamaguchi et al., 2014). Single *Ink4a/Arf* loss has no effect on tumor lesions but leads to their rapid progression to highly invasive and metastatic pancreatic adenocarcinoma in the presence of *KRAS* ^*G12D*^ (Aguirre et al., 2003). In addition to the common precancerous lesions, PanIN, the co-existence of *KRAS* mutations with the *SMAD4*/*DPC4* tumor suppressor gene deletion in mucinous cystic neoplasms (MCNs) may eventually lead to the development of PDAC (Izeradjene et al., 2007). *KRAS* mutation prevent the reversion of ADM development and accelerate pancreatic intraepithelial neoplasia (PanIN), eventually leading to pancreatic adenocarcinoma in both clinical samples as well as animal models (Liou et al., 2015) (Figure 3).

### 4.2 *KRAS* mutation in PDAC metabolic reprogramming

*KRAS* mutation could accelerate PDAC progression depending on, not only the activation of signaling pathways, but also the induction of metabolic reprogramming (Hu et al., 2019). *KRAS* mutation could regulate glucose, amino acid, and lipid metabolism in PDAC cancer cells (Suzuki et al., 2022).

#### 4.2.1 Glucose

Pancreatic cancer cells facilitate glycolysis under adequate oxygen conditions; this is known as the “Warburg effect” (Chang et al., 2022). Glucose is transported into the cell through glucose transporters (GLUTs), which mainly include GLUT1, GLUT2, GLUT3 and GLUT4 (Reckzeh and Waldmann, 2020). The glucose transferred into the cytoplasm by the GLUT is catalyzed to glucose-6-phosphate(G-6-P) by hexokinase (HK) (De Jesus et al., 2022). Then, G-6-P can be converted to fructose-6-phosphate (F-6-P) by phosphohexose isomerase (PGI). Phosphofructokinase (PFK) can catalyze F-6-P to fructose-1,6-bisphosphate (F-1,6-BP) (Shi et al., 2017). F-1,6-BP can be catabolized to glyceraldehyde 3-phosphate (GA3P) and dihydroxyacetone phosphate (DHAP). Next, triose phosphate isomerase (TPI) converts DHAP to triose phosphate (TP). Glyceraldehyde 3-phosphate dehydrogenase (GAPDH) oxidizes GA3P to 1,3-diphosphoglyceric acid (1,3-DPG). 1,3-DPG is catalyzed by phosphoglycerate kinase (PGK) to produce 3-phosphoglyceric acid (3-PG). 3-PG is converted by phosphoglycerate mutase (PGAM) to glycero-2-phosphate (2-PG). 2-PG is dehydrated by enolase (ENO) to produce phosphate enol pyruvate (PEP). After this step, pyruvate kinase (PK) catalyzes the conversion of PEP to pyruvate and adenosine triphosphate (ATP). Then, the final step in glycolysis is that lactate dehydrogenase (LDH) catalyzes the conversion of pyruvate to L-lactate (Ganapathy-Kanniappan and Geschwind, 2013; Abdel-Wahab et al., 2019).

The fasting glucose level is a risk factor for pancreatic cancer occurrence in type 1 and type 2 diabetes patients, indicating that glucose metabolism is involved in pancreatic cancer development (Menini et al., 2020). A study of the United Kingdom Biobank found that people with diabetes were more than three times more likely to develop PDAC than those without diabetes (Sharma et al., 2022). Chronic type 2 diabetes is a risk factor for PDAC, which is also the cause of new onset diabetes. There is also a strong association with genetic mutations reported by PDAC patients (Andersen et al., 2017). In pancreatic cells, high glucose treatment favors the hexosamine pathway (HPB), which increases the level of O-glycosylation in pancreatic cells, leading to an increased level of O-glycosylation of PFK and a decrease in its activity, positive feedback to the HPB pathway. In diabetic patients, high glucose uses the enhanced HPB pathway to enhance O-glycosylation levels of RNM1, a subunit of nucleotide reductase (RNR), in pancreatic cells, leading to a reduction in the dNTP pool and causing DNA damage, which preferentially induces mutation in KRAS^G12D^. The KRAS^G12D^ accelerates the conversion of pancreatic cells to pancreatic intraepithelial neoplasia and again mediates the reprogramming of glucose metabolism (Hu et al., 2019). *KRAS* ^*G12D*^ promotes GLUT1 expression by upregulating SLC2A1, the gene encoding GLUT1. The expression of leukemia inhibitory factor receptor (LIFR) was significantly lower in pancreatic cancer compared to some cancers, but inhibition of *KRAS* mutation upregulated LIFR expression and was accompanied by a decrease in GLUT1 expression. It was found that LIFR can inhibit GLUT1 expression by activating STAT3, while *KRAS* mutation reversed this effect, allowing GLUT1 to be consistently expressed and promoting glycolysis (Liu et al., 2021).According to data from The Cancer Genome Atlas (TCGA) and the analysis of 89 informative PDAC tumor samples, *KRAS* mutation is involved in pancreatic cancer cell aerobic glycolysis (Zhu et al., 2021). PFKFB3/PFK2, a *KRAS* mutation-dependent glycolytic activator, was reported to contribute to PDAC aerobic glycolysis. KRAS^G12D/G12C^ can promote the expression of PFKFB3 and GLUT2 by activating p38γ MAPK signaling. p38γ MAPK can stabilize PFKFB3 by S467, which is the phosphorylation site of PFKFB3. Meanwhile, p-PFKFB3 can interact with GLUT2 and enhance the expression of GLUT2 (Wang et al., 2020).Among the clinical methods for tumor diagnosis, 2-[^18^F] fluoro-2-deoxy-D-glucose PET-CT is used as a relatively common means to assess tumors with standardized uptake values (SUV_MAX_). The tumor-specific “Warburg effect” increases the uptake of 2-[^18^F] fluoro-2-deoxy-D-glucose and the uptake is inversely correlated with the expression level of F-box and WD repeat domain-containing 7 (FBW7). FBW7 is an important molecule for targeting and destroying many cancer proteins, and its functional inhibition is an important driver of carcinogenesis. It was found that overexpression of FBW7 in pancreatic cancer cells activates the c-Myc signaling pathway to target thioredoxin-binding protein (TXNIP), a negative regulator of glucose metabolism, which in turn inhibits glycolysis-related genes, including GLUT1, GLUT4, HK2, LDHA and LDHB. However, *KRAS* mutation can destabilize FBW7 by activating ERK signaling, which ultimately promotes glycolysis and promotes proliferation of pancreatic cancer cells (Ji et al., 2016). The study found that cancer cells carrying *KRAS* mutation was more likely to survive when cultured under low-glucose condition and that this was closer to the *in vivo* microenvironment of PDAC (Yun et al., 2009; Sullivan et al., 2019). Moreover, the level of autophagy in *KRAS*-mutated cancer cells is increased under low-glucose condition. At the same time, the mitochondrial content and total membrane potential of PDAC cells are reduced. Therefore, the level of ROS in mitochondria is also decreased, which reduces the demand for NADPH and ensures that pancreatic cancer cells can continue to proliferate even under low-glucose condition. Both mitochondrial dysfunction and aerobic glycolysis are important features of cancer cells, and *KRAS* ^*G12V*^ can promote mitochondrial dysfunction and facilitate the shift from oxidative phosphorylation to aerobic glycolysis (Hu et al., 2012).To explore the mechanism of the above phenomenon, Humpton, Tet al. found that *KRAS* ^*G12D*^ can upregulate the mRNA and protein expression levels of Nix through activating MEK signaling pathway to induce mitochondrial autophagy, reduce mitochondrial glucose flux, and finally maintain redox homeostasis in PDAC cells (Humpton et al., 2019). The NADPH mentioned here is necessary for cellular maintenance of redox, and NAD(P)H oxidase (NOX) is an enzyme necessary to catalyze NAD(P)H oxidation, of which NAD(P)H oxidase 4 is one. In PDAC, *KRAS* mutation and p16 deletion are also required for its development, and both can collaborate to promote NOX4 expression, NAD(P)H oxidation and the production of the glycolytic substrate NAD + or NADP+, which can increase glycolysis allowing for sustained proliferation of PDAC cells. p16 deletion alone does not promote NOX4 expression, but also requires *KRAS* ^*G12V*^ mutation to upregulate p22phox, a subunit required for NOX4 activation, through activation of NF-κB. The results indicate that NF-κB is the major transcription factor for p22phox promoter binding (Ju et al., 2017). In addition, NAD(P)H is also an important product of the pentose phosphate pathway (PPP) and is an essential molecule for maintaining the conversion of reduced and oxidized glutathione, which can be referred to as the oxidative branch of PPP. *KRAS* ^*G12D*^ was found to increase glucose uptake to promote the production of NAD(P)H and ribose, and the oxidative and non-oxidative branches of PPP do not affect each other. Meanwhile, *KRAS* ^*G12D*^ promotes the hexosamine pathway (HBP) to increase the level of protein glycosylation, and *KRAS* ^*G12D*^ maintains pancreatic cancer by activating MAPK and Myc signaling pathways to upregulate glycolysis-related genes (GLUT1, HK1, ENO1, and LDHA), HBP-related genes (GFPT1), and PPP non-oxidative branch genes (RIA), respectively (Ying et al., 2012). *KRAS* ^*G12D*^ also can promote glycolysis by using paracrine pathways. *KRAS* ^*G12D*^ can promote the expression of type I cytokine receptor complexes (IL2rγ-IL4r⍺ and IL2rγ-IL13r⍺1) in pancreatic cancer cells, which can receive growth factor signals (IL4 and IL13) from invasive TH2 cells. Activated type I cytokine receptor complexes increase glycolytic flux by activating the JAK1-STAT6-Myc signaling pathway to upregulate glycolysis-related genes (HK2 and ENO1) (Dey et al., 2020). Recent study reveals that BZW1 (Basic leucine zipper and W2 domain–containing protein 1) can promote tumor growth by enhancing eIF2a phosphorylation to promote glycolysis in PDAC. However, the relationship between BZW1 and *KRAS* mutation has not been clearly elucidated (Li et al., 2022).

**FIGURE 4:**
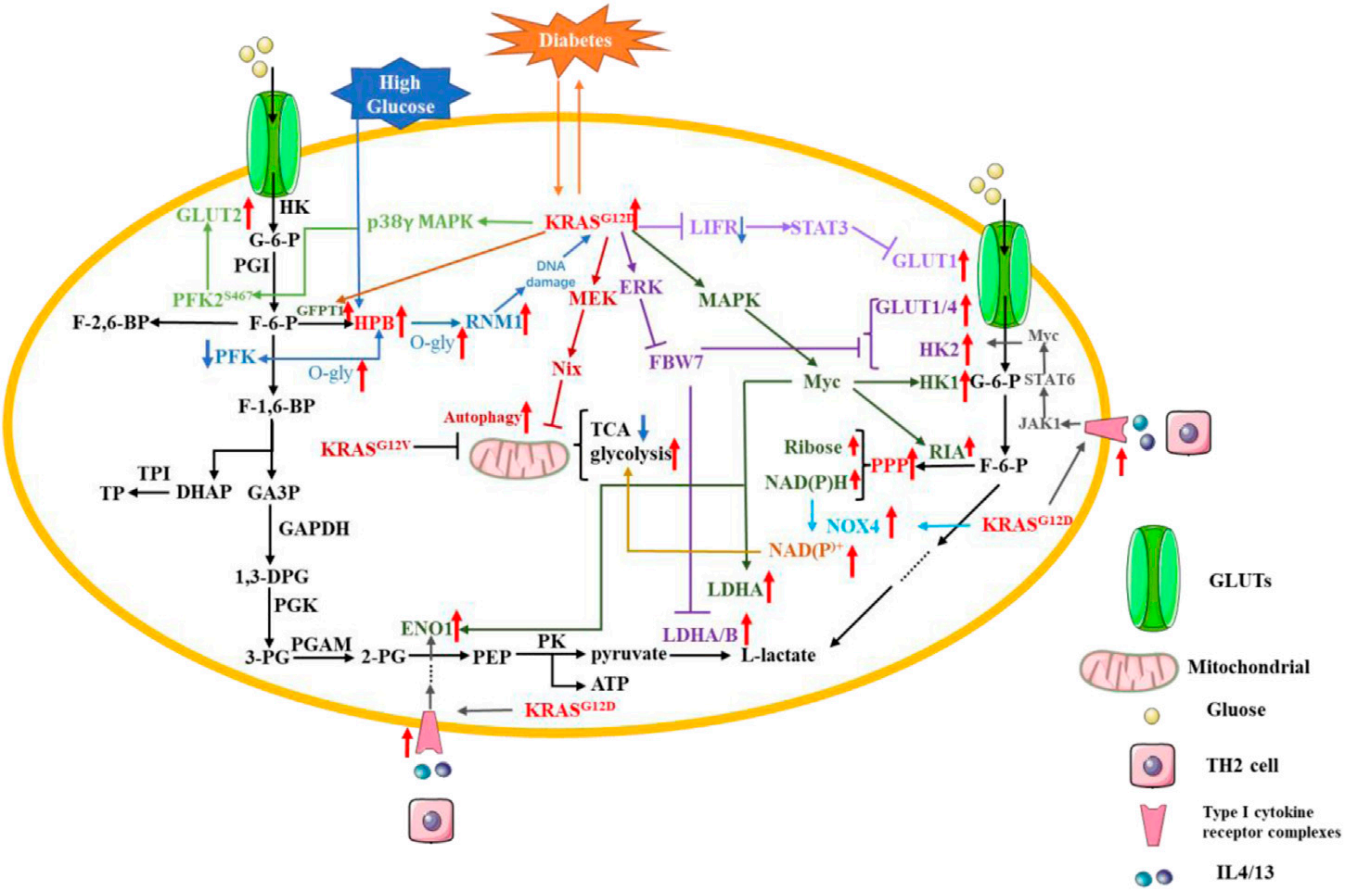
*KRAS* mutation in PDAC glucose metabolic reprogramming. Pancreatic cancer cells facilitate glycolysis under adequate oxygen conditions; this is known as the “Warburg effect. Red arrow: The expression of related gene is upregulated. Blue arrow: The expression of related gene is downregulated.

The use of nanoparticulate albumin bound paclitaxel (Nab-PTX) is one of the more commonly used treatments for pancreatic cancer, however, the RAS/RAF/MEK/ERK signaling pathway inhibits its targeted delivery and effectiveness. The drug efficacy of Nab-PTX was greatly enhanced when glucose deprivation experiments were simulated with an insulin-like growth factor 1 receptor inhibitor (IGF1R) (Li et al., 2021b). Studies have shown that glucose can be an effective, sensitive and inexpensive marker for precancerous lesions of pancreatic cancer. However, the standard threshold for detecting glucose has not been defined and, moreover, it has a low specificity. In contrast, *KRAS* is a highly specific but low-sensitivity biomarker. The combination of the two can greatly improve the detection of precancerous lesions in pancreatic cancer. Moreover, *KRAS* mutation can drive reprogramming of glucose metabolism, and a perfect combination of the two requires a lot of prospective studies before they can truly serve for clinical monitoring (Li et al., 2023) (Figure 4).

#### 4.2.2 Glutamine

In addition to the characteristic “Warburg effect”, glutamine metabolism is another feature of tumor cell metabolism. Furthermore, glutamine is an important substrate for pancreatic cancer biomarkers, CA19-9, and glycosylation (Liu et al., 2023a). Although the “Warburg effect” reduces the availability of pyruvate for the tricarboxylic acid cycle (TCA), glutamine metabolism compensates for this. Glutamine is an alternative nutrient to glucose for PDAC cells as a source of energy and for biosynthesis, especially under glucose limitation conditions (Xu et al., 2022). *KRAS* ^*G12D*^-mutated pancreatic cancer cells were greatly killed when glycolysis-related genes PFKFB3 and glutamine-related GLS1 were simultaneously inhibited (Ozcan et al., 2021). Glutamine, one of the most abundant amino acids in the bloodstream, is a non-essential amino acid, but it provides precursors for the synthesis of a variety of substances, including proteins, lipids and nucleotides, and is an important source of carbon and nitrogen. It also provides essential precursors for the synthesis of NADPH and GSH and maintains redox homeostasis in cancer cells (Cluntun et al., 2017; Zhang et al., 2017; Delgir et al., 2021). Glutamine is transported into the cell via the transporters SLC38A2, SLC38A1, and SLC1A5, which allow the synthesis of hexosamine, aspartate, and nucleic acids in the cytoplasm (Scalise et al., 2017). Glutamine in the cytoplasm must pass through the glutamine transporter SLC1A5 variant to enter the mitochondria. After glutamine enters the mitochondria, glutaminases (GLSs) catalyze the production of glutamate and ammonia, which can be transported to the cytoplasm or remain within the mitochondria. Next, glutamate in the mitochondria is catalyzed by glutamate dehydrogenase 1 (GLUD1) or glutamate transaminase 2 (GPT2) and glutamic acid transaminase 2 (GOT2) to form α-ketoglutarate, which can be transported to the cytoplasm or involved in TCA (Wang et al., 2010; Yang et al., 2017; Stine and Dang, 2020).

**FIGURE 5:**
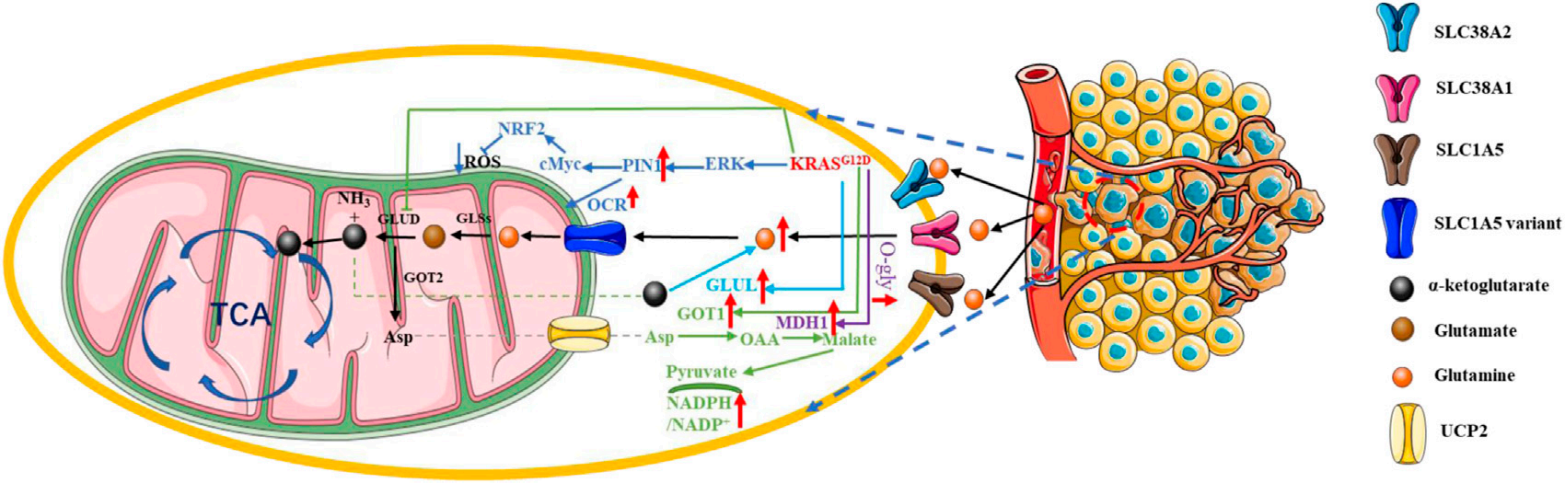
*KRAS* mutation in PDAC glutamine metabolic reprogramming. *KRAS* mutation-containing pancreatic cancer cells have great demand for glutamine; this is also termed as “glutamine addition”. Red arrow: The expression of related gene is upregulated. Blue arrow: The expression of related gene is downregulated.

The “Warburg effect” reduces the availability of pyruvate for the TCA, but glutamine can fuel the TCA cycle by *KRAS* mutation. *KRAS* mutation upregulated Protein interacting with never in mitosis A1 (PIN1) expression to maintain the normal function of mitochondria and redox homeostasis by synergistically activating c-Myc and NRF2, which is necessary for glutamine metabolism (Liang et al., 2019). However, the bloodstream system of pancreatic cancer is poor, and the cancer cells cannot take up enough glutamine. We call this part of cancer cells that do not get enough glutamine as glutamine-deficient cells. *KRAS* mutation-containing pancreatic cancer cells have great demand for glutamine, which is also termed as “glutamine addition”. α-ketoglutarate (*α-KG*) derived from the glutaminase-based catabolism of glutamine provides fuel for the tricarboxylic acid cycle (TCA) in pancreatic cancer cells. Supplementation with *α-KG* restored the growth and survival of glutamine-deficient pancreatic cancer cells with the help of glutamate-ammonia ligase (GLUL), which was elevated in the *KRAS* ^*G12D*^-induced mouse PDAC model (Bott et al., 2019). In most cancer cells, the GLUD1 catalyzed glutamate production of α-ketoglutarate is used as the main fuel for the TCA cycle. In contrast, pancreatic cancer cells can utilize α-ketoglutarate via a non-classical pathway that is mediated by *KRAS* ^*G12D*^ inhibiting GLUD1 and promoting the expression of aspartate transaminase (GOT1). Glutamine-derived aspartate is translocated to the cytoplasm, subsequently, oxaloacetate is converted to malate and pyruvate, increasing the NADPH/NADP (+) ratio and thus maintaining the redox state of pancreatic cancer cells, which makes KRAS-mutated pancreatic cancer cells dependent on this pathway (Son et al., 2013). Thus, inhibition of the aspartate efflux-related protein, mitochondrial transporter uncoupling protein 2 (UCP2), resulted in reduced glutamine hydrolysis and NADPH/NADP+ and glutathione/glutathione disulfide ratios as well as increased levels of reactive oxygen species in *KRAS-*mutated pancreatic cancer cell lines. However, silencing UCP2 just reduced glutamine hydrolysis in wild-type *KRAS* PDAC cells, without affecting their redox homeostasis or proliferation rate (Raho et al., 2020). Moreover, *KRAS* ^*G12D*^ in PDAC cells could also positively regulate the glutamine catabolic pathway by upregulating the *O-*GlcNAcylation of malate dehydrogenase 1 (MDH1), the expression of which was significantly elevated in clinical PDAC samples. This finding demonstrated that *O-*GlcNAcylation may also be related to signaling pathways involving *KRAS* mutation and glutamine metabolism in PDAC (Zhu et al., 2022) (Figure 5).

Recent studies have identified PI3K-C2γ, a class II PI3K, as a negative regulator of PDAC, and its deletion leads to glutamine addiction in PDAC. PI3K-C2γ deletion leads to excessive activation of mTOR signaling, making PDAC glutamine-dependent. However, whether KRAS mutation have a regulatory role in this process has not been clearly elucidated (De Santis et al., 2023). In addition, M2 tumour-associated macrophages can secrete exosomes to promote glutamine metabolism in pancreatic cancer (Liu et al., 2023b). Pancreatic stellate cells can also promote glutamine metabolism in PDAC via the Wnt/β-catenin/TCF7 signaling pathway (Son et al., 2013). *KRAS* mutation promoting glutamine metabolism in pancreatic cancer can also be studied in relation to surrounding stromal cells or associated supporting cells. Glutamine addiction in pancreatic cancer is also a major cause of its chemoresistance (Ganguly et al., 2022; Park et al., 2023). A great deal of research is still needed on glutamine metabolism in pancreatic cancer, which is expected to provide efficient targets for the clinical treatment of pancreatic cancer.

#### 4.2.3 Fatty acids

**FIGURE 6:**
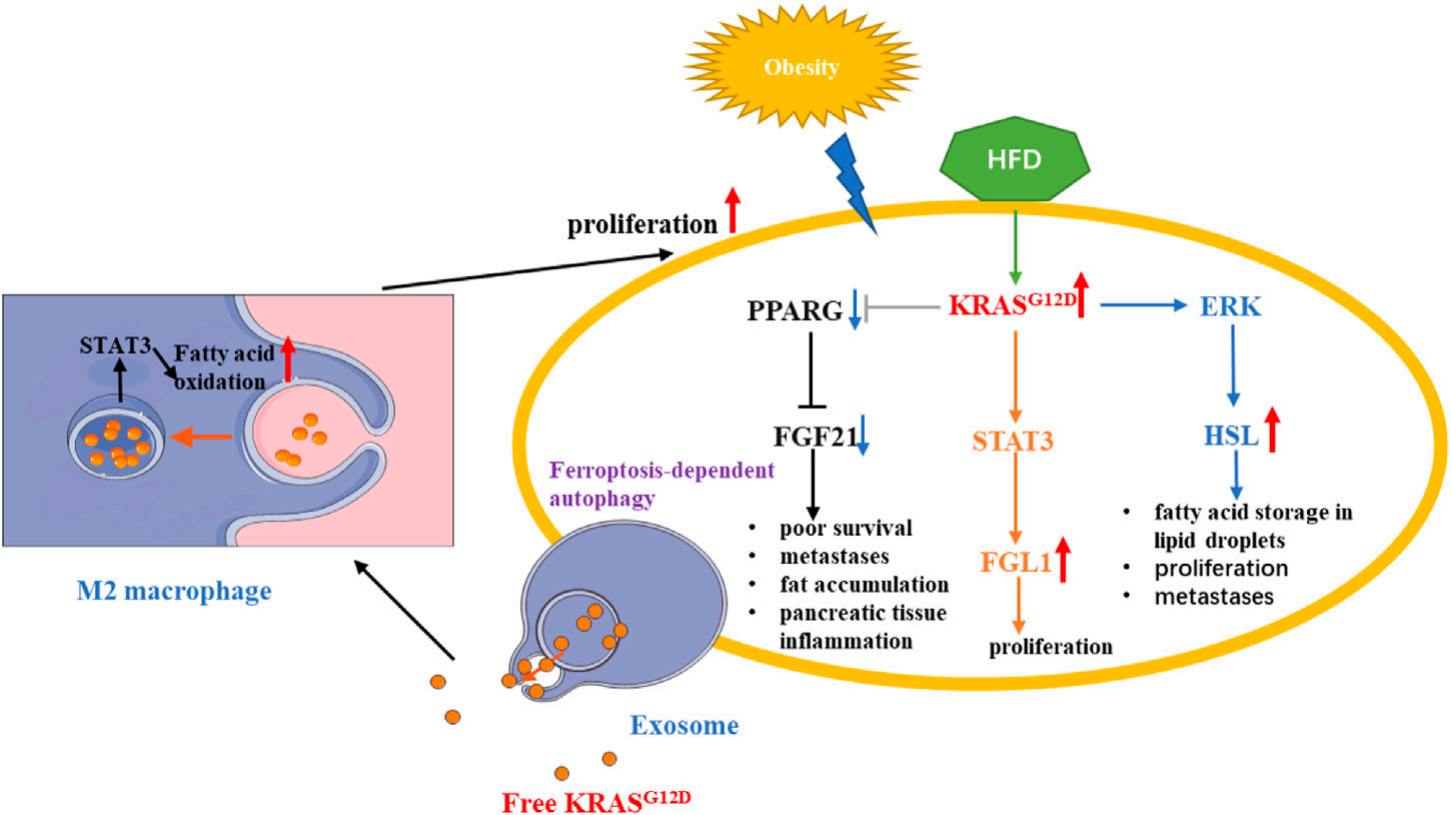
*KRAS* mutation in PDAC fatty acids metabolic reprogramming. *KRAS* mutation could alter lipid metabolism and promote the proliferation of PDAC cells by regulating STAT3/FGL1, FGF21, HSL. In addition, Autophagy-dependent PDAC cells releasing exosome, which contains *KRAS* ^G12D^, can promote fatty acid oxidation by STAT3 in M2 macrophage. Red arrow: The expression of related gene is upregulated. Blue arrow: The expression of related gene is downregulated.

Intraperitoneal fat can infiltrate the pancreas and negatively affect the normal physiological function of the pancreas. Obesity, like diabetes, increases the incidence of pancreatic cancer (Paternoster and Falasca, 2020). Most importantly, Wang, D et al. found that a high-fat diet (HFD), but not a high-carb diet (HCD), induced excessive activation of *KRAS* ^*G12D*^ and further promoted metabolic reprogramming of PDAC (Wang et al., 2019). A high-fat, high-calorie diet (HFCD) increased pancreatic cancer incidence in *P48* ^*+*^ */Cre, LSL-KRAS* ^*G12D*^ (KC) mice, resulting in their exhibition of more extensive inflammation and fibrosis (Chang et al., 2017).A study showed that *KRAS* ^*G12D*^ could upregulate FGL1 through the phosphorylation of STAT3, altering lipid metabolism and promoting the proliferation of PDAC cells (Chiu et al., 2021). As *KRAS* mutation can significantly reduce the expression of FGF21 by downregulating peroxisome proliferator-activated receptor gamma (PPARG), a lipid metabolism activator, injecting HFCD-fed *KRAS* ^*G12D*^/+ mice with recombinant human FGF21 (rhFGF21) could reduce abdominal fat accumulation, triglyceride level, and pancreatic tissue inflammation, ultimately resulting in longer survival and fewer metastases (Luo et al., 2019). *KRAS* mutation also downregulated hormone-sensitive lipase (HSL) by activating ERK signaling pathway to promote lipid storage and excess fatty acid storage in lipid droplets, which are required for pancreatic cancer cell proliferation and metastasis (Man et al., 2020). In addition, free mutant KRAS can also promote pancreatic cancer proliferation through fatty acid metabolism. Ferroptotic pancreatic cancer cells releasing *KRAS* ^*G12D*^ can be taken up by macrophages through an autophagy-dependent mechanism, which polarizes macrophage to an *M2*-like phenotype through *STAT3*-dependent fatty acid oxidation (Dai et al., 2020) (Figure 6).

Recent studies have shown that the lipid-rich lipid raft protein flotillin-1, which can be localized to the cell membrane, interacts with sevenless (SOS)-1 to facilitate the conversion of Ras-bound GDP to GTP. Tissue microarrays from multiple pancreatic cancer samples showed that flotillin-1 was highly expressed compared to normal pancreatic tissue and correlated with *KRAS* (Jin et al., 2023). In addition, the authors found that the SMARCD3, one of SWI/SNF (a nucleosome remodeling complex) subunits, is highly expressed in pancreatic cancer and can synergistically regulate lipid and fatty acid metabolism with FOXA1. Whether it is associated with *KRAS* mutation is also an unsolved mystery (Ferguson et al., 2023).

#### 4.2.4 Acetyl coenzyme A

Histone H4 acetylation levels were elevated in pancreatic follicular cells containing *KRAS* ^*G12D*^ prior to the appearance of precancerous lesions and this elevation was caused by the upregulation of acetyl coenzyme A by *KRAS* ^*G12D*^, which can promote ADM via the mevalonate pathway and thereby promote pancreatic carcinogenesis and epithelial modifications (Carrer et al., 2019). Acetyl coenzyme A synthase short chain family member 2 (ACSS2) is an acetyl coenzyme A synthase. ACSS2 promotes macropinocytosis and facilitates metabolic reprogramming of pancreatic cancer in pancreatic cancer (Zhou et al., 2022). In human PDAC cells, *KRAS* mutation has been shown to promote macropinocytosis, which is an essential strategy for nutrient uptake by cancer cells in the form of macromolecules and cellular debris (King et al., 2020). However, it is not known whether ACSS2 is regulated by *KRAS* mutation.

#### 4.2.5 BCAA

BCAA levels in booldstream are associated with an increased risk of PDAC in pancreatic cancer (Rossmeislová et al., 2021). Branched-chain amino acid (BCAA) metabolism has also been implicated in the development of PDAC. *KRAS* ^*G12V*^ could stabilize *BCAT2*. Tissue-specific knockdown of *BCAT2* in the pancreas can hinder the progression of PanIN in *LSL-KRAS* ^*G12D*^ */+; Pdx1-Cre* (*KC*) mice. A low-BCAA diet was also found to hinder the development of PDAC in mouse models. Thus, BCAA-mediated catabolism is critical for the development of *KRAS*-mutated PDAC (Li et al., 2020). Acetylation of BCAT2 promotes the degradation of BCAT2, which inhibits the cell proliferation of PDAC (Lei et al., 2020). In addition, BCAT1 promotes branched chain ketoacid addiction in mesenchymal-rich PDAC cells and pancreatic stellate cells can also promote pancreatic cancer progression by regulating BCAA metabolism (Zhu et al., 2020; Jiang et al., 2021). Whether *KRAS* mutation can affect BCAA metabolism in pancreatic cancer mesenchymal cells has not been studied in detail.

#### 4.2.6 H_2_O_2_

Early studies have confirmed that reactive oxygen species (ROS), especially hydrogen peroxide(H_2_O_2_), play an important role in maintaining cellular and tissue homeostasis (Park et al., 2021). However, some studies have highlighted that low levels of ROS are more able to promote the progression of pancreatic cancer with *KRAS* ^*G12D*^ mutation (Melzer et al., 2022). Some studies have in turn emphasized that reducing ROS production delays the progression of pancreatic cancer with *KRAS* ^*G12D*^ mutation (Eser et al., 2014; Bear et al., 2020; Ohara et al., 2022). The exact role of ROS in the cell is not clear (Hou et al., 2020). Recently, a mass spectrometry-based study found that the oncogenic *KRAS* ^*G12D*^ mutation promotes the accumulation of H_2_O_2_, but not superoxide or hydroxyl radicals, in pancreatic cancer cells. The *KRAS* ^*G12D*^ mutation inhibits the expression of the catalase gene (CAT) as a way to reduce the breakdown of H_2_O_2_. Because most oxygen radicals are unstable in the cell, being able to measure it precisely is difficult, so this finding is great. The findings suggest that excessive accumulation of H_2_O_2_ can modify intracellular metabolites, including lipids, deoxycholic acid and palmitoylcarnitine, to maintain the continued survival of pancreatic cancer cells (Ferguson et al., 2022). When exogenous H_2_O_2_ is added, the KRAS/AMPK signaling pathway is activated and promotes increased expression of glycolytic genes and lactate production. H_2_O_2_ can increase the flux of glycolysis (Tang et al., 2021). However, one question that while excess H_2_O_2_ promotes pancreatic cancer proliferation, its inevitable effects on unsaturated lipids, such as phospholipid-rich cell membranes (Choi et al., 2019), induce ferroptosis in pancreatic cancer cells (Zhang et al., 2022). However, an answer to this query was soon available, and recent studies have shown that *KRAS* ^*G12D*^ can activate the MAPK signaling pathway, which in turn further activates NRF2, which in turn upregulates the expression of FSP1 (ferroptosis suppressor protein 1) and removes excess ROS, allowing pancreatic cancer cells to proliferate stably under safe conditions (Deramaudt and Rustgi, 2005). In summary, *KRAS* mutation can promote the proliferation of pancreatic cancer cells through a perfect collaborative regulatory pathway.

### 4.3 *KRAS* mutation promotes pancreatic cancer cells immune escape

**FIGURE 7:**
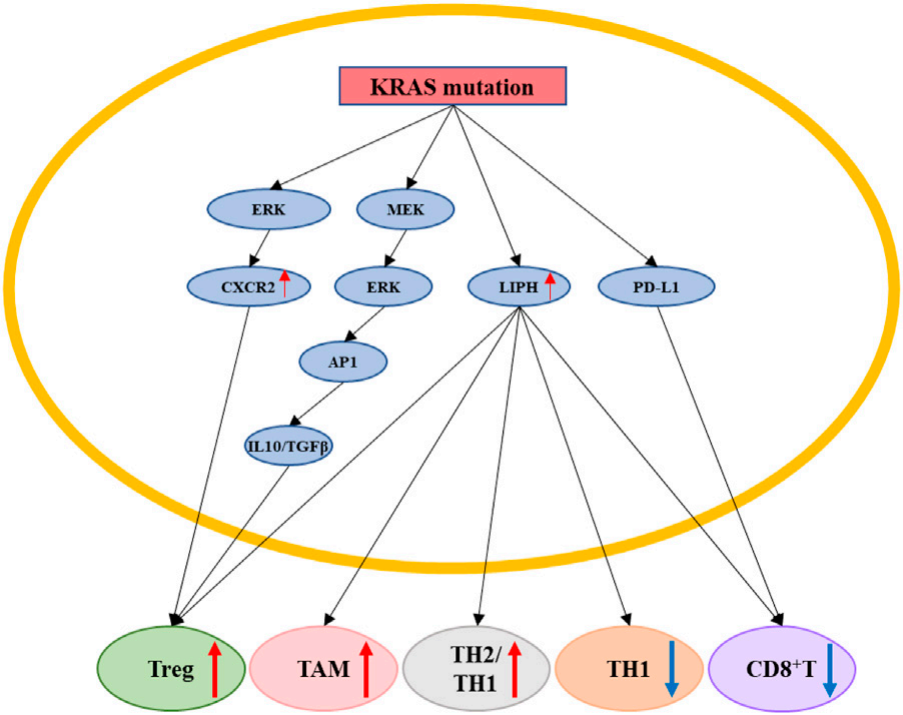
*KRAS* mutation promotes pancreatic cancer cells immune escape. *KRAS* mutation can indirectly affect pancreatic cancer cells by regulating the tumor immune microenvironment by regulating ERK/CXCR2, MEK/ERK/AP1/(IL10&TGFβ), LIPH, PD-L1. *KRAS* mutation could upregulate the proportion of Treg, TAM, TH2/TH1 cells, and downregulate the proportion of TH1, CD8+T cells. Red arrow: The expression of related gene is upregulated. Blue arrow: The expression of related gene is downregulated.

In addition to affecting pancreatic cancer cells biology in an intrinsic manner, *KRAS* mutation can also indirectly affect pancreatic cancer cells by regulating the tumor microenvironment (TME). *KRAS* mutation can suppress T-cell number and function in pancreatic cancer cells, while silencing *KRAS* can reverse this effect. *KRAS* ^*G12D*^ was found to promote pancreatic cancer progression by upregulating the expression of the chemokine *CXCR2* through the *ERK* pathway and thereby enhancing the infiltration of immunosuppressive cells into the TME (Purohit et al., 2016). *KRAS* ^*G12V*^-overexpressing pancreatic cancer cells were also found to enhance regulatory T cell (Treg) activity. In addition, the KRAS-MEK-ERK-AP1 signaling pathway induced PDAC cells to secrete abundant amounts of *IL-10* and transforming growth factor-*β1* (Zdanov et al., 2016), which are essential for maintaining Treg proliferation and function. *KRAS* mutation upregulated lipase *H* (*LIPH*) expression, enhancing the infiltration of tumor-associated macrophages (TAMs), Treg cells, and Th2/Th1 cells into the TME, while reducing the infiltration of CD8+ T cells and Th1 cells; this further contributed to tumor recurrence, worsening of histological grade, and poorer overall survival (Zhuang et al., 2022). The effectiveness of currently existing immune checkpoint inhibitors for treating *KRAS* mutation-containing pancreatic cancer is disappointing and indicates the need to explore additional pathways involved in immune evasion that should be targeted for therapy. Surprisingly, *KRAS* mutations were found to dysregulate cell death pathways, which are induced by the tumor necrosis factor (TNF) receptor. *KRAS* mutations in pancreatic cancer cells rely on both endogenous *TNF*-related apoptosis-inducing ligands (TRAILs) as well as their receptors to promote tumor growth and metastasis (von Karstedt and Walczak, 2020). Interestingly, *KRAS* mutations evaded immune therapy by upregulating programmed cell death 1 ligand 1 (PD-L1) transcription and protein expression (Coelho et al., 2017). *KRAS* mutation could also enhance the recycling of PD-L1 molecules in pancreatic cancer cells to the cell surface to promote the immune escape of pancreatic cancer cells (Hashimoto et al., 2019). High N6-methyladenosine (m6A) expression was associated with reduced PDAC immune infiltration and T-cell depletion, poor overall survival, and tumor recurrence. Moreover, *KRAS* mutation-containing PDAC tumors displayed high m6A scores, suggesting a correlation between *KRAS*, m6A, and immune escape (Zhou et al., 2021) (Figure 7).

### 4.4 *KRAS* mutation in pancreatic cancer therapy resistance

Targeting oncogenes, such as *KRAS*, *NRG1*, and *NTRK*, and their related molecules for therapy is always a hot topic in the tumor research field. However, most results of such research have been disappointing (Qian et al., 2020)*. KRAS* is the most common oncogene in PDAC and targeting it has been very challenging, with *KRAS* mutation-containing PDAC long being considered undruggable. Most existing therapies for this cancer have focused on indirect targeting and have shown limited efficacy (Bannoura et al., 2021) (Table.1). In advanced stage PDAC, *KRAS* mutation upregulates the expression of the antioxidant factor *NRF2* in response to higher oxidative stress levels in PDAC cells, which leads to chemoresistance (Mukhopadhyay et al., 2020). Several specific *KRAS* mutation inhibitors have been developed, most of which target *KRAS* ^*G12C*^. However, the *KRAS* ^*G12C*^ mutation can only be detected in a small fraction of PDAC patients (Nagasaka et al., 2021). In addition, patients with *KRAS* ^*G12C*^ mutation-containing PDAC respond poorly to *KRAS* ^*G12C*^ inhibitors, mainly because: 1) *KRAS* ^*G12C*^ co-exists with other *KRAS* point mutations or at a high copy number; 2) the presence of other mutant genes besides *KRAS*; 3) accelerated PDAC metastasis and novel genetic variations, such as *MET* amplification; *NRAS*, *BRAF*, *MAP2K1*, *RET*, *ALK*, *RET*, RAF1, and *FGFR3* gene fusions; and loss of *NF1* and *PTEN* tumor suppressor function (Nussinov et al., 2021). *KRAS* mutation not only drive the development of PDAC but also contribute to PDAC tumor heterogeneity during progression. Gemcitabine (GEM), a first-line PDAC chemotherapeutic drug, has limited efficacy in most patients, which may be due to *KRAS* mutation (Won et al., 2021). In one study, *KRAS* mutation-activated PI3K/AKT/mTOR and AXL signaling contributed to pancreatic cancer cell resistance to MEK (a *KRAS* effector) inhibitors (Pettazzoni et al., 2015). However, combining MEK inhibitors with a PI3K/mTOR inhibitor still failed to cure PDAC in the presence of *KRAS* ^*G13D*^ (Neuzillet et al., 2014). Although the combination of MEK inhibitors with standard therapy (gemcitabine/Nab-paclitaxel) reduced tumor growth and metastasis in PDXs (patient-derived xenograft models), it may also elicit *KRAS* mutation, which can contribute to tumor heterogeneity. *In vitro* and *in vivo* analyses suggested that MEK inhibitor-induced PDAC heterogeneity facilitates the rapid proliferation of treatment-resistant cells (Pedersen et al., 2017). Related data show that *CasRx* can precisely and effectively silence the expression of mutant *KRAS* ^*G12D*^ in PDAC cells and, further, eliminate *KRAS* ^*G12D*^-related downstream signaling, thereby, making PDAC cells sensitive to gemcitabine and inhibiting tumor growth (Jiang et al., 2020).

**TABLE 1:** Therapies for PDAC directly focused on *KRAS* mutation or indirectly on related effector.

Drug	Target	Rationale	Study phase	Ref
LC-2	Mutant *KRAS*	Degradation of *KRAS* ^G12C^	Preclinical	Bond et al. (2020)
17f	Mutant PDEδ	Degradation of PDEδ	Preclinical	Cheng et al. (2020b)
Borussertib	AKT	Inhibiting the proliferation *KRAS*-mutant pancreatic cancer	Preclinical	Weisner et al. (2019)
TCR gene therapy	*KRAS* ^G12D^	Inhibiting *KRAS* ^G12D^	Preclinical	Leidner et al. (2022)
36I	PDEδ	inhibiting *KRAS*-PDEδ interaction	Preclinical	Chen et al. (2022)
RMC-6291, RMC-6236	Mutant *KRAS*	Binding to *KRAS* and preventing *KRAS*-mediated signaling	Preclinical	Dougherty et al. (2019)
KD2 cyclic peptide	Mutant *KRAS*	Inhibiting the GTP-bound form of mutant *KRAS* ^*G12D*^	Preclinical	Zhang et al. (2020)
KS-58 bicyclic peptide	Mutant *KRAS*	Cyclic peptide binding mutant KRAS G12D	Preclinical	Sakamoto et al. (2020)
BI-3406	The interaction between *KRAS* and SOS1	A Potent and Selective SOS1-*KRAS* interaction inhibitor	Preclinical	Hofmann et al. (2021)
MRTX1133	Mutant *KRAS*	Binding *KRAS* ^*G12D*^	Preclinical	Kemp et al. (2023)
*KRAS* peptide vaccine	Mutant *KRAS*	Long peptide vaccine for KRAS-mutant pancreatic cancer	Phase I trial (NCT04117087)	(Ju et al., 2017), (Frank et al. (2022))
mDC3/8-KRAS vaccine	Dendritic cell vaccine	Targeting *KRAS-* mutant resectable PDAC	Phase I trial (NCT03592888)	
RMC-4630/LY3214996	SHP2/ERK	Good tolerability and efficacy of the combination for *KRAS*-mutant pancreatic cancer	Phase I trial (NCT03634982) (NCT04916236)	
CCT3833	RAF/SRC	Inhibiting both RAF and SRC in *KRAS*-mutant pancreatic cancer	Phase I trial (NCT02437227)	Saturno et al. (2021)
MK-8353	ERK1/2	Disrupting ERK dimerization or binding to the active conformation	Phase I trial (NCT01358331) (NCT03745989)	Roskoski (2019)
AT5719	CDK1/2/7/9	Inhibiting the phosphorylation of CDK1/2/7/9 substrates in mutant *KRAS* -driven pancreatic cancer	FDA approval	Kazi et al. (2021)
Palbociclib	CDK4/6	Impairing recovery from cytotoxic chemotherapy in PDAC with *KRAS* ^G12V^	FDA approval	Salvador-Barbero et al. (2020)
Sotorasib/MRTX849	*KRAS* ^G12C^	Targeting the *KRAS* ^G12C^	FDA approval	Hong et al. (2020)
Trametinib/Tuxolitinib	MEK/STAT3	Reprogramming the cancer-associated fibroblast (CAF) and immune microenvironment to overcome resistance to immune checkpoint inhibition in PDAC	FDA approval	Datta et al. (2022)

### 4.5 *KRAS* mutation in pancreatic cancer diagnosis and prognosis

As one of the most devastating digestive system cancers, PDAC is characterized by a lack of specific symptoms, fast progression, and poor prognosis (Chen et al., 2021a). Combining the identification of *KRAS* mutation and cytopathology increased the sensitivity, accuracy, and negative predictive value of pancreatic cancer diagnosis and molecular subtype classification. Compared to conventional serum biomarkers and medical imaging, examining the *KRAS* mutation level using genomic and transcriptomic techniques has significantly improved diagnostic sensitivity and specificity, indicating potential clinical application value (Lundy et al., 2021). In addition, the presence of *KRAS* mutations in the blood circulation was positively associated with poorer prognosis (Buscail et al., 2020). Moreover, *KRAS* ^*G12V*^ mutations in the circulation of patients with PDAC was associated with a high percentage of circulating Tregs. Both, the presence of high *KRAS* ^*G12V*^ mutations and Tregs in the circulation in PDAC, indicate advanced disease stage, extremely poor survival, and poor response to immunotherapy (Cheng et al., 2020a). Compared to clinical serum markers, circulating *KRAS* mutant *DNA* levels showed more sensitivity and specificity in predicting the effect of chemotherapy on tumor mass burden. Kruger et al. (2018) found that *KRAS* mutant *DNA* levels decreased immediately upon treatment of patients with PDAC with gemcitabine-based treatment, while there were no significant changes in the DNA levels of *CA 19–9*, *CEA*, and *CYFRA 21–1* until 4 weeks after treatment; this indicated that *KRAS* mutant *DNA* is an early indicator of gemcitabine-based chemotherapy response. Repeated measurements of *KRAS* mutant DNA levels act as a superior marker for PDAC progression (sensitivity: 83%, specificity: 100%). In addition to chemotherapy, radiation therapy is also commonly used in pancreatic cancer treatment. Blocking *KRAS* mutations made pancreatic cancer tumors sensitive to radiation therapy both *in vivo* and *in vitro* (Brunner et al., 2005) (Allenson et al., 2017).

Local and distant metastasis indicate a poor prognosis of PDAC patients. The *KRAS* mutant DNA level in circulation was significantly consistent with metastasis to tissues in patients with advanced PDAC, indicating that the circulating *KRAS* mutant DNA level may be a potential biomarker to predict metastasis in PDAC (Patel et al., 2019). *KRAS* ^*G12D*^ activated human heterogeneous cytosolic ribonucleoprotein A1 (HnRNPA1) in a ubiquitin-like protein (SUMO2)-dependent manner, promoting its export from cancer cells to the extracellular space in a vesicle-mediated (EV-mediated) manner as well as promoting lymphangiogenesis and lymph node metastasis. Mechanistically, a study revealed that *KRAS* ^*G12D*^ induced HnRNPA1 hyperactivation, interacted with TSG101, and enhanced hnRNPA1 packaging and delivery via EVs, which were internalized by human endothelial lymphocytes in the tumor microenvironment and promoted lymph node metastasis (Luo et al., 2022). Through genomic, transcriptomic, methylomic, and molecular dynamics analyses, the *KRAS* mutant and the stability of the protein expressed by it could predict the poor prognosis of pancreatic cancer (Kaushik et al., 2021). In a mouse PDAC model, *KRAS* mutations promoted tumor-associated fibroblast heterogeneity, which resulted in pancreatic cancer cell metastasis and poor prognosis. Pan et al. (2021) found that tumor cells associated with metastasis-induced inflammatory CAFs (iCAFs) were with heterogeneity. CAFs from liver metastases exhibited a more homogeneous phenotype, while CAFs from lung metastases remained heterogeneous. Thus, it was difficult to implement a uniform approach to treat pancreatic cancer with a satisfactory survival rate. Compared to common PDAC, 73% adenosquamous carcinoma of the pancreas (ASCP) cases were found to involve *KRAS* mutations using whole-genome copy number variation (CNV), whole-exome sequencing, and transposase-accessible chromatin by sequencing (ATAC-seq), which may be the reason for higher malignancy and poorer prognosis in ASCP than in PDAC (Lenkiewicz et al., 2020).

## 5 Conclusion

The involvement of *KRAS* mutations has been widely researched in pancreatic cancer cell signaling activation, metabolic reprogramming, immune escape, therapy resistance, and prognosis. Although some *KRAS* mutation inhibitors have been used in clinical practice, they have shown few effects in the treatment of patients with PDAC. Due to the absence of specific symptoms, most *KRAS* mutations in PDAC are identified and studied in the advanced stage of the disease. Thus, the exact role of *KRAS* mutations in early stage PDAC is still unclear.

Review of existing literature in this area indicates that researchers should focus their attention on studying the role of *KRAS* mutations in early stage of pancreatic cancer to improve the early screening rate and, thereby, prolong the patient survival rate and improve the life quality in pancreatic cancer. This can be done through two ways. 1) The use of advanced research tools, such as organoids, patient-derived xenograft (PDX) models, and genetically engineered mouse models (GEMMs) for PDAC. However, due to the heterogeneous nature of pancreatic cancer cells, immune-deficient mice used to create PDX models lacked an effective immune microenvironment and pancreatic genetic diversity. Therefore, they could not completely simulate the pancreatic cancer TME and its response to treatments (Chen et al., 2021b). Although the establishment of an organoid requires an advanced organ culture model to form a pancreatic niche *in vitro* from the porcine bladder (PUB) (Melzer et al., 2022), it cannot acquire better experiment repeatability and confirm that every cell contained a genetic mutation, such as *KRAS*. Thus, to accurately simulate the pancreatic cancer microenvironment, a pancreatic model that contains stable *KRAS* mutations and can reveal the development of additional *KRAS* point mutations after the introduction of a certain treatment is required. Using such a model, one could better investigate the role of *KRAS* mutations in the early stages of pancreatic cancer. 2) The application of nanomaterials is another promising method for studying the role of *KRAS* mutations in early stage of pancreatic cancer. Traditional drug delivery methods have failed to distinguish cancer cells from normal cells (Conde et al., 2016). Nanomaterials could be an efficient medium for delivering si*RNA* to knock out *KRAS* (Mehta et al., 2019) from cells. However, these nanomaterials may elicit an immune response and, therefore, be hard to use in clinical practice. Thus, a nanomaterial that does not elicit an immune response from the human body is required. In addition, this nanomaterial should be able to modify various *KRAS* mutations and, therefore, act as a unified therapy.

## Figures and Tables

**Table:** 

**KRAS-WT**	the wild type of KRAS
**KRAS-MUT**	the mutation of KRAS
**GAP**	GTPase activating protein
**GDP**	Guanine ribonucleoside diphosphate
**GTP**	Guanine ribonucleoside triphosphate
**GEF**	Guanine nucleotide exchange factor
**PI3K**	Phosphatidylinositol 3-kinase
**AKT**	protein kinase B
**mTOR**	Mechanistic target of rapamycin
**RAF**	Serine/threonine protein kinase
**MEK**	Mitogen-activated protein kinase kinase
**ERK**	extracellular signal-regulated kinase
**RALA/RALB**	v-ral simian leukemia viral oncogene homolog A/B
**PLC**	phospholipase C
**PDK1**	pyruvate dehydrogenase kinase
**MYC**	myelocytomatosis oncogene
**CDK5**	Cyclin—dependent Kinase 5
**YAP**	Yes-Associated Protein
**JAK**	Janus Kinase
**STAT3**	Signal transducer and activator of transcription 3
**AURKA**	The mitotic kinase aurora A
**TPX2**	Targeting protein for *xenopus* kinesin-like protein 2
**GATA6**	Zinc finger transcription factor 6
**EGFR**	epi-dermal growth factor
**ALK4**	Anaplastic lymphoma kinase 4
**ATDC**	ataxia capillaries group D complementary gene
**PFKFB3**	6-phosphofructo-2-kinase/fructose-2,6-biphosphatase 3
**LIF**	Leukemia inhibitory factor
**GLUT1**	Glucose transporter 1
**Nix**	Alias
**BNIP3a**	adenovirus E1B 19 kDa interacting protein 3-like
**FBW7**	F-box and WD repeat domain-containing 7
**JAK1**	Ja-nus Kinase 1
**STAT6**	Signal transducer and activator of transcription 6
**cMyc**	c-myelocytomatosis oncogene
**NOX4**	NADPH oxidase 4
**GLUL**	glutamate-ammonia ligase
**NRF2**	Nuclear factor erythroid 2-related factor
**PIN1**	Protein NIMA-interacting 1
**MDH1**	malate dehydrogenase 1
**FGL1**	Fibrinogen-like 1
**FGF21**	Fibroblast growth factor 21
**HSL**	Hormone-sensitive lipase
**CXCR2**	Chemokine (C-X-C motif) receptor 2
**AP1**	Jun proto-oncogene
**IL10**	Interleukin 10
**TGFβ**	Transforming growth factor β
**LIPH**	lipase H
**PD-L1**	Programmed cell death 1 ligand 1
